# Assessing contemporary Arctic habitat availability for a woolly mammoth proxy

**DOI:** 10.1038/s41598-024-60442-7

**Published:** 2024-04-29

**Authors:** Jessie Poquérusse, Casey Lance Brown, Camille Gaillard, Chris Doughty, Love Dalén, Austin J. Gallagher, Matthew Wooller, Nikita Zimov, George M. Church, Ben Lamm, Eriona Hysolli

**Affiliations:** 1 Colossal Biosciences Inc, Austin, TX 78701 USA; 2 School of Informatics, Computing, and Cyber Systems, Northern Arizona University, Flagstaff, AZ 86011 USA; 3 Department of Zoology, Stockholm University, Stockholm, Sweden; 4 Centre for Palaeogenetics, Svante Arrhenius Väg 20C, Stockholm, Sweden; 5 Department of Bioinformatics and Genetics, Swedish Museum of Natural History, Stockholm, Sweden; 6 Beneath The Waves, Boston, MA 02129 USA; 7 College of Fisheries and Ocean Sciences, University of Alaska Fairbanks, Fairbanks, AK 99775 USA; 8 North-East Science Station, Pacific Institute of Geography, Russian Academy of Sciences, Chersky, Russia; 9 Wyss Institute for Biologically Inspired Engineering, Harvard University, Boston, MA 02115 USA; 10 Department of Genetics, Harvard Medical School, Boston, MA 02115 USA; 11 Harvard-MIT Program in Health Sciences and Technology, Cambridge, MA 02139 USA

**Keywords:** Carrying capacity, Biomass, Remote sensing, Woolly mammoth proxy, Ecological engineering, Ecology, Ecology

## Abstract

Interest continues to grow in Arctic megafaunal ecological engineering, but, since the mass extinction of megafauna ~ 12–15 ka, key physiographic variables and available forage continue to change. Here we sought to assess the extent to which contemporary Arctic ecosystems are conducive to the rewilding of megaherbivores, using a woolly mammoth (*M. primigenius*) proxy as a model species. We first perform a literature review on woolly mammoth dietary habits. We then leverage Oak Ridge National Laboratories Distributive Active Archive Center Global Aboveground and Belowground Biomass Carbon Density Maps to generate aboveground biomass carbon density estimates in plant functional types consumed by the woolly mammoth at 300 m resolution on Alaska’s North Slope. We supplement these analyses with a NASA Arctic Boreal Vulnerability Experiment dataset to downgrade overall biomass estimates to digestible levels. We further downgrade available forage by using a conversion factor representing the relationship between total biomass and net primary productivity (NPP) for arctic vegetation types. Integrating these estimates with the forage needs of woolly mammoths, we conservatively estimate Alaska’s North Slope could support densities of 0.0–0.38 woolly mammoth km^−2^ (mean 0.13) across a variety of habitats. These results may inform innovative rewilding strategies.

## Introduction

### Herbivorous megafaunal Arctic ecological engineering

Interest continues to grow in trophic rewilding as a climate change mitigation strategy^1,2^, and Arctic herbivorous megafaunal ecological engineering in particular^3^.

Herbivores share a long co-evolutionary history with vegetation^4,5^ and stimulate plant production in grasslands^6^. The “keystone herbivore” hypothesis stipulates that megaherbivores have maintained the Pleistocene’s steppe-tundra (also referred to as “mammoth steppe”) through complex biophysical (e.g. trampling and uprooting) and biogeochemical interactions^7,8^. Modified vegetation architecture and species composition, increased albedo and therefore cooling, long distance seed dispersal, and enhanced nutrient cycling/productivity and carbon storage each represent a special class of scalar effects primarily active when megaherbivores are present^9^.

A wave of megafauna extinctions took place ~ 12–15 ka, coinciding with arctic shrub tundra expansion and a loss of the Pleistocene steppe-tundra. It remains debated to what extent the change in vegetation was a top-down^10–12^ result of human-precipitated megafaunal extinctions or the megafaunal extinctions a bottom-up^13,14^ response to coeval climate change (e.g. increased moisture/temperatures). Ultimately, only 7 of the 13 megafauna taxa present in eastern Beringia immediately prior to ~ 12–15 ka survived, which left an impoverished ecological community.

Megafaunal biomass today is estimated to be about 30 times less dense than in the Pleistocene^15^ and complex networks of biophysical and biogeochemical interactions are currently missing in much of the Arctic biome. This means that vast swaths of land they used to inhabit have been replaced by forests and shrubs rather than the forb- and graminoid-rich grasslands for which they were ecologically tuned and possibly played a key role in maintaining the presence and productivity of.

In addition, in recent decades, amplified warming trends continue to modify Arctic landscapes, characterized by coincident decreases in lichens and graminoid biomass and increases in deciduous and evergreen shrub biomass^16^. Satellite remote sensing has revealed a resultant widespread increase in overall plant productivity and biomass across the Arctic tundra biome^17^. These changes are only exacerbated by the continued evidence of rapid ecological shifts to novel ecosystems resulting from continued contemporary megaherbivore extinctions^18^.

The impacts of megaherbivores on grassland preservation and productivity may point to a contemporary natural climate solution of reverting the current wet/moist moss and shrub-dominated tundra and the sparse forest–tundra ecotone to grassland through the rewilding of a guild of large herbivores in the Arctic, the extent of which remains unknown.

### Rationale

Resolving the theoretical carrying capacity of contemporary Arctic ecosystems to rewilded megaherbivores is critical to planning for such megafaunal ecological engineering strategies of meaningful impact^19^.

Since vegetation landscapes have changed in the last ~ 12–15 ka, we sought to assess whether and to what extent contemporary Arctic landscapes were conducive to the rewilding of megaherbivores, using a woolly mammoth (*M. primigenius*) proxy. Since woolly mammoths thrived in steppe-tundra, boreal, and temperate habitats across the northern circumpolar region approximately 700,000 to ~ 10,000 years ago (with the very last populations going extinct 4000 years ago)^20^, we use them as a model species^21,22^.

Previous modeling work has pointed to an estimated total megaherbivore density during the Late Pleistocene ranging from 10.5 tonne km^−2^ (2.5 tonnes km^−2^ for woolly mammoths) based on the density of bone remains in Northern Siberia^21^ to 8.8 tonne km^−2^ total megafauna (4.5 tonnes km^−2^ for woolly mammoths) based on assumed caribou (*R. tarandus*) density comparisons^15^.

In this study, we sought to provide a novel estimate of the carrying capacity of megaherbivores of contemporary Arctic regions. We specifically leverage global meta-analyzed biomass carbon density data at a 300-m resolution to resolve the conservative carrying capacity of a population of rewilded megaherbivores on the North Slope of Alaska using a woolly mammoth proxy, hereafter referred to as woolly mammoth, as a model species. To this end we assess the biomass of their primary forage (forbs, graminoids, and deciduous shrubs) across the North Slope, downgrading it to take into account yearly primary productivity only, and compare it to their intake needs based on body mass estimates.

## Results

### Review of the preferred forage types of the woolly mammoth

Despite harboring evolutionary adaptations that facilitate grazing, woolly mammoths were monogastric herbivorous generalists^23^ with a high degree of flexibility in their food choice, engaging in mixed-feeding outside core feeding areas with a preference for grazing^24^. This is consistent with the fact that mammoths had high *δ*^15^N values (which are higher in grasses than shrubs) than modern elephants, as determined by isotopic analyses of woolly mammoth bone collagen^25^. Their foraging habits are further aligned with the tendency of megaherbivores to have generally long gut retention times, exploit resources across broad spatial scales, and utilize mixed feeding strategies to survive on lower quality, seasonally-restricted vegetation sources^26^. Further, the megaherbivore body size itself likely evolved to process diverse low-quality forage while surviving harsh climates^27^.

A combination of independent lines of evidence including palynological (pollen) analyses of gut content^28^, multiproxy analyses of dung from the lower intestine (including microscopic, chemical, and molecular techniques such as gas chromatography/mass spectrometry, thermally assisted hydrolysis and methylation, and DNA sequencing)^29^, visual analyses of gut content^30^, fatty acid profiling of fatty tissues^31^, DNA sequencing / metabarcoding of gut and coprolite samples^32^, meta-proteomic analyses by shotgun mass spectrometry of gut tissue samples^33^, DNA metabarcoding, palynological and macrofossil analyses of coprolite (feces) samples^34^, and isotopic analyses of tooth enamel^35,36^, suggest that woolly mammoths consumed primarily forbs, graminoids (grasses and sedges), and shrubs, supplementing their diet with trees mosses and even lichen and green algae (as detailed in Supplementary Table 1).

Nutrient-dense forbs were a staple^32^, while potentially large graminoids (reaching up to 75–100 cm), may have also provided ample nutritional value^34^. In addition, herbivores in the Arctic have a strong preference for palatable deciduous shrubs compared with evergreen shrubs when given the choice, which, as supported by gut and coprolite content studies, stands to reason would have likely been the case for the woolly mammoth as well^37,38^. Only trace amounts of trees were reported in previous studies, which are unsubstantial compared to the contribution of forbs, graminoids, and deciduous shrubs (as reported in Supplementary Table 1). In addition, mosses were not expected to play a critical role in their diets given their low digestibility and nutritional value. Moss has been suggested to be consumed in cold environments exclusively for their high concentration of arachidonic acid which helps animals protect against the cold^39^, and the consumption of moss by muskoxen has been suggested to indicate low availability of favored foods when confined to small winter ranges^40^. Meanwhile, even though herbivores like reindeer and caribou are known to consume large quantities of low-protein lichens during winter^41,42^, very little is known about their relative contribution to the woolly mammoth diet. Finally, only one study mentioned algae, which was therefore omitted from the woolly mammoth diet in our model.

These dietary habits, despite environmental disparities, are relatively aligned with those of the woolly mammoth’s closest living relative the Asian elephant (*E. maximus)*, a mixed browser and grazer^43^ with a proclivity for a variety of grasses, shrubs, and some trees^44^. Additionally, mixed feeding by elephants appears related to seasonal availability and preference, a trend seen in other arctic ungulates (as detailed in the “Discussion”)^45^.

These foraging habits likely contributed to their high degree of mobility revealed by multiple isotopic analyses^20^. Over their estimated life span of 60 years^46^, the large-scale, geographically unrestrained movements of an individual woolly mammoth were complex^20^ and could exceed a radius of 200 km^36,47^.

However, paleoecological habits should be placed into the context of certain ongoing ecological changes that could affect the future diets of woolly mammoth. These include the northward advance of the tundra shrubline^48^, changing seasonal climates^49^, and changes in fire regimes^50^.

For our model, in keeping with our objective of establishing a cautious, conservative estimate for a woolly mammoth carrying capacity in the contemporary Arctic, we deliberately concentrated our analysis exclusively on the plant functional types favored by woolly mammoths for which we found the most empirical evidence, specifically forbs, graminoids, and deciduous shrubs.

### Carrying capacity estimates based on downgraded, mean digestible annually generated biomass

Collectively, the percent cover, reflecting variations in AgDB across biomes, and digestibility factors, reflecting variations in the estimated digestibility of the woolly mammoth’s preferred forage types, resulted in downgrading factors ranging from 0.0 to 0.62 (mean 0.15).

**Figure 1:**
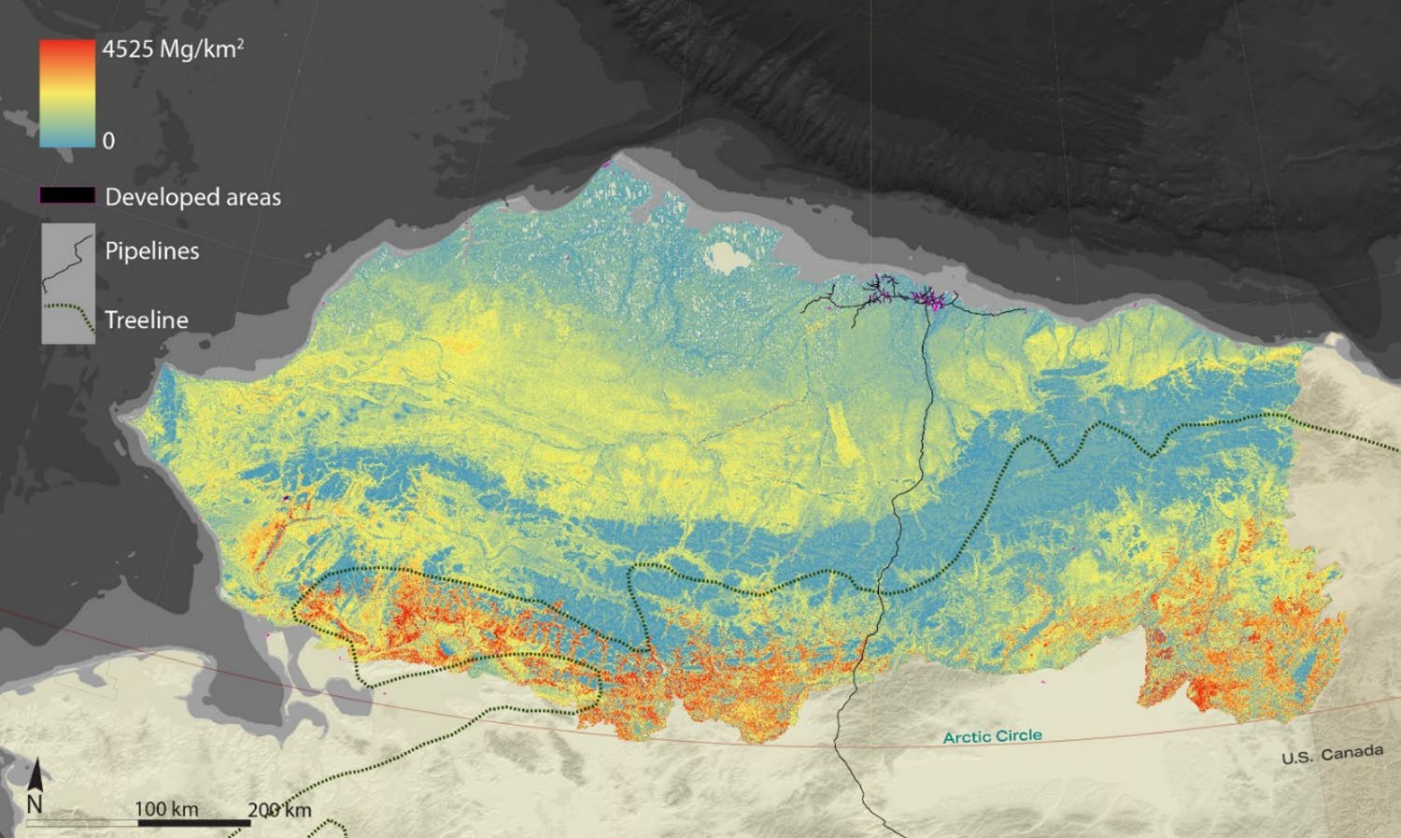
Downgraded, mean digestible annually generated biomass of woolly mammoth preferred plant functional types (forbs, graminoids, deciduous shrubs) in the North Slope of Alaska. Map created in Esri ArcGIS 10.3.1 (https://support.esri.com/en-us/products/arcmap) and text/legends in Adobe Illustrator 28.3 (https://www.adobe.com/products/illustrator.html).

Higher downgrading factors are concentrated in the southern section where higher shrub biomass occurs (as visualized in Supplementary Fig. 1 and Fig. 1). The latitudinal banding pattern of the ecoregions remains legible in the AgDB results, with higher readings in the uplands sections of the foothills and the southernmost boreal sections. The coastal plain and mountains sections show the lowest AgDB readings. The downgrading process preserved the overall pattern reported by Berner et al. of roughly double biomass in the warmer southern section of this area compared to the colder, northern sections.

The estimated AgDB of ecological landscapes generally increases with its mean average annual temperature. Arctic and boreal rocky uplands, due to their large spatial extents and above average AgDB have the largest overall carrying capacity followed by both Arctic/boreal rocky acidic alpine units, while the Arctic silty uplands rank third.

**Figure 2:**
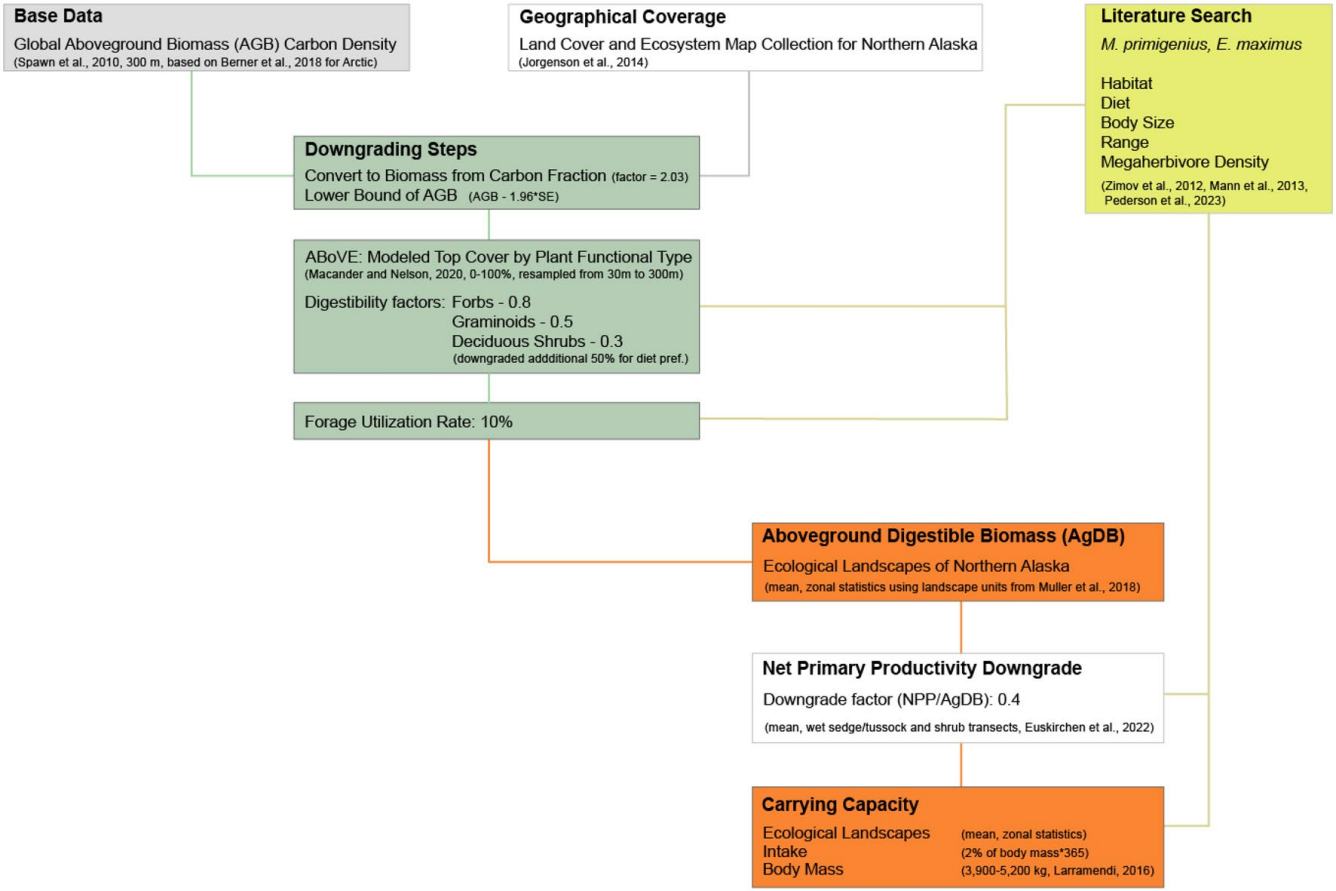
Conceptual diagram of the downgrading process leading to the carrying capacity estimates.

Finally, the net primary productivity downgraded resulted in an additional crude Arctic-specific downgrading factor of 0.4 over the entire area (see Materials and Methods for details, and Fig. 2 for a conceptual diagram of the complete downgrading process).

**Figure 3:**
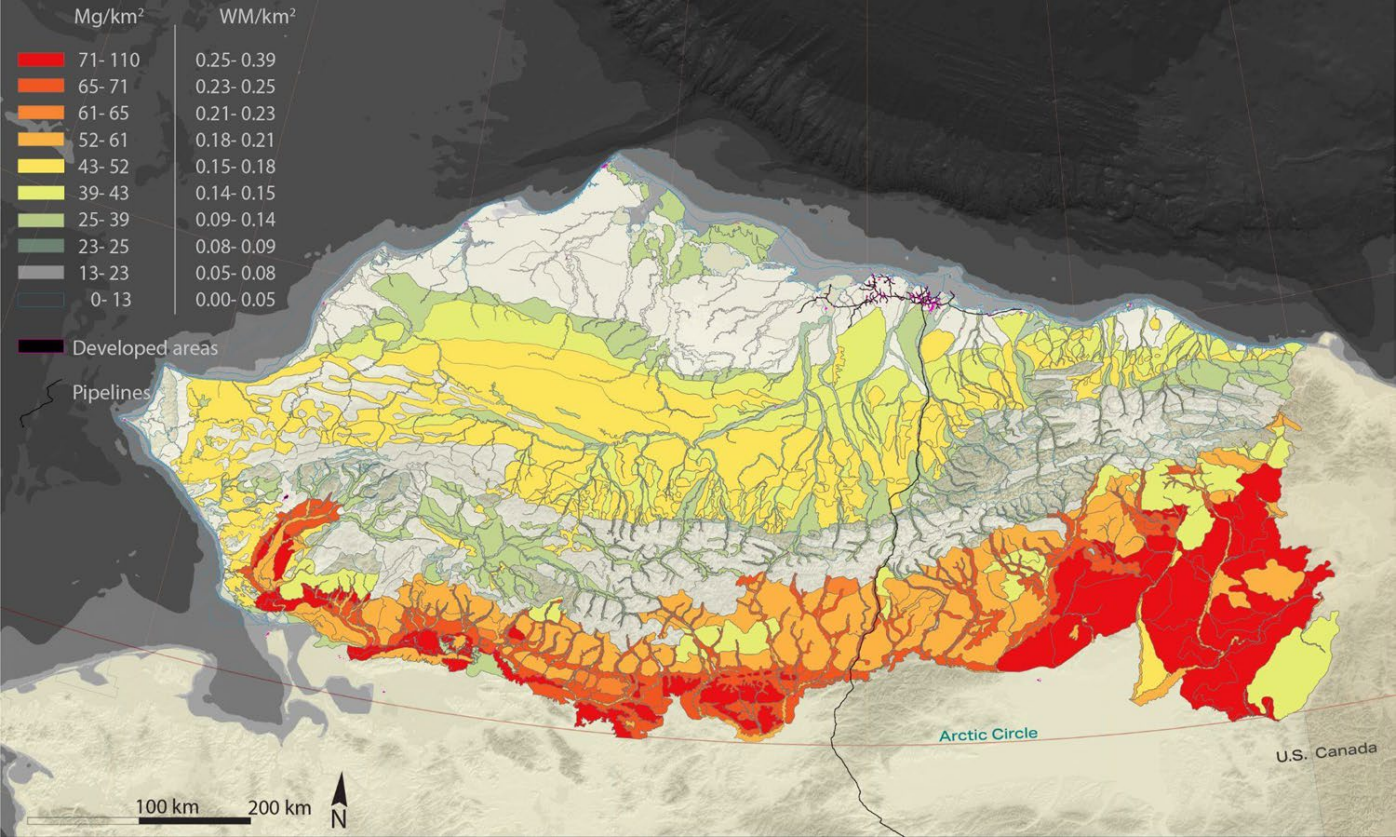
Estimated mean digestible annually generated biomass of woolly mammoth preferred plant functional types (forbs, graminoids, deciduous shrubs) available for forage and derived woolly mammoth carrying capacities in the North Slope of Alaska. Specifically, the legend’s left column represents the total digestible annually generated biomass of woolly mammoth-preferred plant functional types (forbs, graminoids, deciduous shrubs) (Mg km^−2^) while the legend’s right column represents the derived woolly mammoth carrying capacity based on their estimated annual forage needs based on a woolly mammoth body mass of 3.9 tonnes (see Supplementary Table 2 for details). Map created in Esri ArcGIS 10.3.1 (https://support.esri.com/en-us/products/arcmap) and text/legends in Adobe Illustrator 28.3 (https://www.adobe.com/products/illustrator.html).

Considering the annual forage consumption of a woolly mammoth, using these final mean digestible annually generated biomass density estimates, we calculate that the ecological landscapes of Northern Alaska could support populations of 0.0–0.38 woolly mammoth km^−2^ (mean 0.13) across a variety of habitats (as visualized in Fig. 3), reflecting the diversity of the landscape of the study area, and ranging from a low of 0.0 in fresh and marine water landscapes to a high of 0.29–0.38 in Boreal Sandy Riverine (taiga) landscapes (as detailed in Supplementary Table 2). Across all ecological landscapes, these density estimates suggest that the entire North Slope of Alaska could, in the absence of predators, major losses from disease, and landscape exclusions, among other considerations, theoretically support ~ 48,000 woolly mammoths (~ 42,000 for an adult woolly mammoth body mass of 5.2 tonnes, to ~ 55,000 for an adult woolly mammoth body mass of 3.9 tonnes).

### Population density estimates based on Damuth’s law

We further sought to complement our analyses by using the known allometric relationship between population density and body mass, i.e. Damuth’s law, whereby population density is scaled with body mass raised to the power of − 3/4 irrespective of taxa or time^51^. Applying Damuth’s law, whereby log D =  − 0.75 (log W) + 4.23, with D the population density (km^−2^) and W the mean adult body mass of a mammalian primary consumer (in grams), yields, under the assumption of an adult woolly mammoth body mass ranging between 3.9 and 5.2 tonnes, a population density of ~ 0.16–0.19 individuals km^−2^. Across all ecological landscapes, these density estimates suggest that the entire North Slope of Alaska could theoretically support ~ 72,000 woolly mammoths (~ 64,000 for an adult woolly mammoth body mass of 5.2 tonnes, to ~ 80,000 for an adult woolly mammoth body mass of 3.9 tonnes).

### Summary of previously and presently carried out estimates

Finally, we summarize in Table 1 previously carried out estimates and the estimates carried out in this paper using both a net primary productivity-based model as well as a classical allometric scaling law in ecology.

**Table 1:** Pleistocene and contemporary woolly mammoth carrying capacity estimates across Arctic latitudes. Woolly mammoth km^−2^ estimates were derived, where not explicitly provided, under the assumption of a mean woolly mammoth body mass of 4550 kg (see “Discussion” for a discussion of body mass estimate)^46^.

Previously carried out estimates	Method	Region	Period	Total woolly mammoth biomass km^−2^	Woolly mammoth km^−2^
Zimov et al.	Population density calculated based on observed bone remains^54^	Northern Siberia	Pleistocene	2.5 tonnes	0.55
Mann et al.	Population density estimated based on assumed caribou density comparisons^15^	Arctic Alaska	Pleistocene	4.5 tonnes	0.99
Pederson et al.	Population density modeled as a function of body mass using phylogenetically adjusted allometric models^52^	Not applicable	Not applicable	7.7 tonnes	1.7
**This paper’s estimates**					
Net primary productivity-based model	Carrying capacity estimated using WM-specific forage net primary productivity	Alaska North slope	Contemporary	0.0–1.8 tonnes	0.0–0.38
Damuth’s law-based	Population density estimated applying classical theoretical ecology law^51^	Not applicable	Not applicable	0.8 tonnes	0.17

## Discussion

### Woolly mammoth body mass estimate

We selected to use an estimated woolly mammoth body mass from an in-depth study by Larramendi et al. on the height, body mass, and shape of fully grown adult proboscideans across different taxa using a variety of skeletal restorations^46^. From this study we used the estimated body mass of an average weight *M. primigenius* male of the North Siberian form (3.9–5.2 tonnes), considered a closer estimate of an Arctic-specific body mass than the European form, meaning that across the different ecological landscapes of the North Slope of Alaska, the different ranges of woolly mammoth densities are thus based on these lower (3.9 tonnes) and higher (5.2 tonnes) body mass estimates, resulting, respectively, in the density estimates’ higher and lower bounds. Our estimated body mass range is roughly consistent with the PHYLACINE 1.2 global database of late Quaternary mammals trait data, which, also leveraging Larramendi et al.’s estimates, defined the mean woolly mammoth mass at 6 tonnes, without distinguishing between the European or North Siberian form^52,53^. Since very little is known about the demographic structure of woolly mammoths, including sex, age, and reproductive-to-non-reproductive ratios, we did not explicitly account for variations due to the body masses of females or young. Importantly, using the average body mass of an adult male, by yielding a more conservative carrying capacity estimate, is aligned with our methodological priorities.

### Consistency of carrying capacity estimates with previous estimates

More convincingly, our estimates for woolly mammoth carrying capacity are in line with previous estimations of woolly mammoth densities during the Pleistocene (as detailed in Table 1). Previous efforts to characterize woolly mammoth densities range from 0.55 woolly mammoth km^−2^ (10.5 tonne km^−2^ total megafauna biomass, 2.5 tonne km^−2^ woolly mammoth biomass; in Northern Siberia based on skeleton densities of woolly mammoths^54^) to 0.99 woolly mammoth km^−2^ (8.8 tonne km^−2^ total megafauna biomass, 4.5 tonne km^−2^ woolly mammoth biomass; in Arctic North America, based on caribou analogue^15^). Finally, leveraging the model of a recent study which modeled population densities for extant and recently extinct mammals using field metabolic rates^52^ yielded an estimated woolly mammoth density of 1.7 woolly mammoth km^−2^. These models may best represent some physiological optimization of megaherbivores and their ecologically-engineered habitat, which the Arctic is currently missing.

Our carrying capacity, based on a net primary productivity-based model, of 0.0–0.38 woolly mammoth km^−2^ is the same order of magnitude and vast portions of the ecological landscapes match some previous estimates (e.g. mean of all Arctic landscapes could support ~ 0.13 woolly mammoth km^−2^). However, mismatches in estimates may be due to the limitations inherent to our process, the vegetation changes between the Pleistocene and modern-day, or previously carried out estimates being biased due to over/underrepresentation of skeletal remains in the Siberian deposits, inaccurate assumption of stable caribou densities since the Pleistocene, or incorrect assumptions of body mass.

### Conservative nature of estimates

Our estimates are conservative on a number of independent fronts. (1) Our choice to omit from the woolly mammoth diet evergreen shrubs and trees, as well as mosses, lichen, and algae yield more conservative carrying capacity estimates by virtue of our under-representation of the full breadth of the true woolly mammoth diet. This is because there is insufficient information to assess their dietary contributions, (2) There is more plant biomass in the Arctic available today than when the data we analyzed was obtained (2010), and projections estimate that shrub cover in the Arctic could increase by 2- to tenfold over the next 50 years^48^. The taxonomic diversity of arctic plant assemblages is also greater now and continues to rise^32^. (3) Forbs, which are more nutrient-dense than other woolly mammoth nutrient sources^34^, may be underrepresented in the Anthropocene Arctic where niche construction by megaherbivores is absent. We also used the highest estimated body mass/nutritional needs and the lowest grazing rates. (4) In the future, climate-induced increases in snowfall over the North Slope, resulting in expanded growing seasons, with correspondent increases in leaf nitrogen content, digestible protein and foraging period are expected to affect the body mass/fat reserves in caribou^55^. In addition, by blocking snow and light from reaching the ground, the expansion of shrubs may increase nitrogen and foliar biomass on some species, ultimately increasing forage quality and quantity^56^. Consequently, we would expect woolly mammoths to encounter a climatic shift-associated increase in biomass availability.

By opting for a pure endogenous biomass approach however, without incorporating any exogenous parasitic, predatorial, fire/disturbance, or competition dynamics^57^, our measures do not include certain factors that could further reduce carrying capacity.

### Woolly mammoth flexibility and adaptability

#### Woolly mammoth dietary diversity

A striking aspect of the woolly mammoth diet was its remarkable diversity in composition and spatial breadth. Molar enamel profiles show diets alternating between browse and grasses likely dependent on seasonal and/or geographic availability, similar to contemporary proboscideans^32,45,58^. Woolly mammoth guts have been found to harbor the seeds of herbaceous species and genera common to both tundra and boreal zones^59^, and recent multiproxy analyses have suggested that woolly mammoths occupied a variety of environments in addition to the cold, dry “mammoth steppe”, spanning wet, marshy environments, saline meadows, dry meadows, and even gravely slopes in mountainous/rocky habitats^34^. On St Paul island, a study from Wang et al. (Ref^60^ discussed more below) has suggested that woolly mammoths can survive on just shrub and graminoid tundra in the Holocene era. This is consistent with the fact that woolly mammoths had high *δ*^15^N values, which are higher in grasses than shrubs, as determined by isotopic analyses of woolly mammoth bone collagen, compared to modern elephants^25^. High *δ*^15^N_Phe_ values of the woolly mammoth further imply that within these habitats they were able to selectively consume plants more enriched in ^15^N than the forage consumed by most other herbivores^61^.

This is aligned with recent work pointing to the mosaic and dynamic nature of the Pleistocene “mammoth steppe”, which, likely recourse partitioning within their ecosystems^25^, woolly mammoths could fully exploit given their flexible, varied diets from different non-analogous habitats^34^ spanning their vast geographic distribution^62^. Further, the broad classification of the Palearctic landscape as “mammoth-steppe” may misrepresent a more complex environment with wet, mesic, and woodland ecotypes present in northern Eurasia and North American during and after the Pleistocene^63^.

Furthermore, different woolly mammoths as a result of intraspecific variation were likely to have different energy economies in terms of both composition and volume^64^, demonstrating a high degree of flexibility and adaptability.

Consistently, caribou and other ungulate studies show constant influence of snow and ice constraints on winter feeding^65^, shifting from selective when resource-abundant to energy-restricted and quantity focused later. Indeed, recent descriptions of caribou diet reveal a dynamic diet influenced by seasonal variations in resource availability, with a preference for graminoids and vascular plants in warmer seasons. In addition, during population declines, females adjust their habitat use, favoring shrublands and barrens with greater plant resources^66^, overall demonstrating a remarkable degree of habitat and dietary adaptability^42^.

In a similar fashion, during droughts, modern African elephants constrict their feeding ranges to areas with permanent water and increase their reliance on woody vegetation, as a result of decreased grass availability^67,68^. Metcalfe et al.’s results suggest that woolly mammoths in Arizona’s San Pedro Valley sought the greenest portions of the landscape, as do modern elephants^35,69^.

#### Unique dietary niches between sympatric species

Although the isotopically defined dietary niche of woolly mammoths and mastodons shows increasing overlap as they approach extinction implying a certain degree of competition^36^, isotopic analyses of bulk collagen suggest that mammoths occupy a distinct habitat or forage niche relative to other Pleistocene herbivores^61^.

### Ecodividends: ecosystem feedback loops

The “keystone herbivore” hypothesis suggests that megaherbivores would have been key to unintendedly geo-engineering and maintaining the Pleistocene’s steppe-tundra through a number of complex biophysical and biogeochemical interactions^7,8,70,71^, which, pending further studies specifying these impacts, point to important potential impacts of a rewilded population of megaherbivores in contemporary Arctic regions. First, as evidence of positive feedforward loops between the growth of grasslands and presence of megaherbivores continues to grow^72,73^, polyphage woolly mammoths may have maintained a grass/herb ecosystem by uprooting shrub/tree assemblages^7,74^—not only contributing to an increased surface albedo but also helping explain the high productivity of the Pleistocene’s steppe-tundra^27,75^. Second, snow removal and trampling by megaherbivores may lead to a more condensed, colder layer of permafrost; data suggests that Pleistocene park proxies like caribou, Yakutian horses, and bison can increase soil heat diffusion and decrease soil temperatures by 1.9 °C compared to control areas^76^. Third, megaherbivores dispersed the seeds of the plants they consumed, enhancing the species richness and adaptability of plant communities while acting as vectors of nutrient movement^77^; effects are only expected to increase under the increased soil nutrient conditions expected with continued Arctic warming^78^. Finally, data from yak and ibex in a Trans-Himalayan ecosystem^79^, muskoxen in Greenland^80^, and various herbivores in Northern Siberia suggest that soil carbon pools are stabilized by the presence of mammalian herbivores, leading to shallower thaw depths and enhanced carbon storage compared to non-grazed regions^81^.

#### Trophic rewilding: an optimistic future

Concretely, the rewilding of other proboscideans, such as the African elephant, has proved successful in recent years, enhancing the open, mosaic nature of their ecosystems^82^, while the trophic rewilding of ungulates has reduced woody invaders at landscape scale in Mozambique in a mere 4-year period in Gonrongosa^83^.

Meanwhile species distribution models have been used to suggest that future climate change might not prevent megafauna rewilding^84^, and increased attention is thus duly being paid to trophic rewilding, or ‘the restoration of missing ecological functions and evolutionary potential of lost megafauna’, as a climate mitigation strategy^1^. More broadly, this is reflective of a new thinking that supports the restoration and conservation of wild animals and their ecosystem roles as a key element of natural climate solutions^1,2,85^.

### Limitations: assumptions and approximations

#### No perfect extant analogs for woolly mammoth habitat or physiology

While a collagen isotope study showed that environmental proxies for Pleistocene steppe might be landscapes found in west-central Yukon, eastern Kluane Lake, and south-central Whitehorse valley^86^, no intact, largescale analogs exist^75^. Others have pointed to the plateaus and basins found in the Altai-Sayan Mountains in southern Siberia as a potential relict with long surviving Late Pleistocene animals, plants and an overall steppe-tundra mosaic landscape^87^. In addition, it remains challenging to accurately use physiological models for large endotherms, especially extinct ones, as there remains a high degree of unknowns in their biodata^88^. Our selected downgrading factors for preferred forage types, digestibility rates, and forage utilization rates, although based on studies of other herbivores, may not perfectly represent woolly mammoth dietary preferences; applying our 50% reduction for shrubs based on proboscidean dietary preferences for example involves a hereto unresolved assumption about woolly mammoth behavior.

#### Pleistocene vs. contemporary Arctic plant composition

Several lines of evidence suggest that plant distributions have not substantially changed since the Pleistocene across the North Slope of Alaska. Several grasses and forbs still exist in the tundra; their relative prevalence have been marginally altered by a set of biotic (e.g. megafaunal pressure) and abiotic factors^89^. In addition ancient environmental DNA from the Yukon further indicates that no plant taxa became locally extinct during the transition to the Pleistocene-Holocene transition^90^. Rather, the relative dominance of graminoids/forbs likely flipped with shrubs/forest/woody species. Overall however, the complexity and variability of ecosystems over time, including the changing Arctic landscape due to climate change, land cover change, and other factors, make it exceptionally challenging to accurately predict the precise impact of reintroducing extinct megaherbivores.

#### Plant digestibility, palatability, non-toxicity

In addition to physical defenses and adjusting growth rate, plants produce chemicals that can optimally, in response to herbivore pressure, decrease their digestibility, palatability, or increase their toxicity^91–95^. However, herbivores adapt their dietary strategies accordingly. In muskoxen (*O. moschatus*) (ruminants), season and diet are known to affect fiber digestion by relying on a large suite of bacterial genera to digest plants that vary widely in both abundance and nutritional quality throughout the year^96^. Meanwhile, caribou avoid over half of understory vegetation, possibly because of low digestibility and defensive compounds of mosses, evergreen shrubs, and conifers^38^. Various herbivorous/omnivorous megafauna are further known to develop seasonal diet mixing and macronutrient optimization strategies, as has been demonstrated in the grizzly bear (*U. arctos*) for example^97^.

We used conservative downgrading factors to approximately account for remaining unknowns in plant digestibility and palatability, and woolly mammoth adaptability thereto.

#### Seasonal variations

In our model, we use an annually averaged NPP estimate, assuming a constant relationship between aboveground biomass and net primary productivity, and annually averaged dry matter intake estimate of 2%. This incorporates a certain degree of uncertainty since our model is limited by unknowns related to varied metabolic needs and dry matter intake of certain populations, such as reproductive females, and winter forage availability. Yet, the woolly mammoth and arctic herbivores are highly adaptable in their diet and tend adjust their metabolic needs according to the season. In the Arctic, while summers allow for a replenishment of body reserves, long 9–10 month winters have low food and increased energy demands (for net energy maximization), for which caribou, elk, bison, muskoxen, Przewalski horses, and woolly mammoths are known to physiologically adapt through reduced resting time, reduced organ size, slowed metabolic rate, minimized forage time, slowed gut passage time, and increased fat storage^31,98–100^. This complexity presents challenges modeling existing arctic mammals, and even more so an extinct non-ruminant herbivore, which is why we selected to use a conservative large-bodied herbivore estimated dry matter intake of 2%. The precise impacts of winters however continue to warrant additional research.

#### Interspecies competition

The true carrying capacity of woolly mammoths on the North Slope of Alaska will further depend on the level and direction of competition between woolly mammoths and extant arctic species. Despite our use of conservative downgrading factors (including foraging factor), there remain unknowns with regard to the impacts on and of such interspecies competition dynamics. For example whether woolly mammoths were more or less efficient competitors relative to caribou and small mammals (e.g. lemmings, arctic ground squirrels) remains unknown and may affect our woolly mammoth carrying capacity estimates. In addition, the dynamic and interconnected nature of modern ecosystems are not entirely reflected in our straightforward model. In particular, the potential interactions with current species, vegetation dynamics, and land use practices may affect woolly mammoth carrying capacities; these elements may be addressed in future research.

## Conclusion

Trophic rewilding triggers a range of complex, interrelated questions of compatibility, capacity, and resilience. Though the present study only begins to delve into that complex set of interacting conditions, megaherbivore capacity is critically dependent on the digestible biomass available for foraging intake. We provide a technically feasible density range for woolly mammoths based on a conservative set of nutritional and behavioral assumptions which are roughly one order of magnitude lower than paleontological estimates of woolly mammoth densities (2.5–4.5 tonne km^−2^). Naturally, a variety of real world limitations paired with a complex set of ecological feedbacks will net out a more refined supportable megaherbivore density.

Here we emphasize that the rewilding of de-extinct functional proxies for ecological engineering purposes prompts important philosophical considerations^102–104^. The ability of this strategy to restore lost ecosystem functionality needs to be balanced with the potential unintended consequences of reintroducing extinct species into more contemporary environments and the allocation of resources to de-extinction alongside ongoing conservation efforts of existing species and habitats. De-extinct megafaunal ecological engineering represents one innovative conservation tool of many^101^, which must be married to ongoing conservation efforts. In so doing, any planned reintroduction effort must be informed by multi-stakeholder conversations about ecological, political, and animal and human welfare concerns with local communities and regional and federal legislators^105^. To this end, the International Union for the Conservation of Nature (IUCN)’s 2016 guidelines for de-extinction practice^106^ continue to guide a dynamically evolving regulatory framework emphasizing the importance of interdisciplinary dialogue and inclusive governance structures.

Ultimately, building on this theoretical carrying capacity of keystone herbivorous megafauna like the woolly mammoth could advance reintroduction strategies, enhance overall biodiversity initiatives, and prioritize largescale ecological engineering feedbacks throughout the formerly occupied megafaunal ranges.

## Methods

### Literature review of the preferred forage types of the woolly mammoth

The peer-reviewed literature was combed for high-yield information on woolly mammoth dietary habits by performing a Boolean operator-guided literature search in PubMed and Semantic Scholar for “woolly mammoth AND (diet OR nutrition OR forage)”).

We qualitatively complemented these analyses with knowledge of the woolly mammoth’s closest living relative, the Asian elephant (*E. maximus*).

### Woolly mammoth dietary needs estimates

Graphic Double Integration volumetric estimates for *M. primigenius* of the North Siberian form suggest a mean body mass for an adult male ranging from 3900 to 5200 kg, slightly heavier than *E. maximus*^46^.

To assess daily dietary needs, we use the broadly applicable 2% body mass rule, whereby a species’ daily nutritional needs in biomass (kg) is estimated at 2% its body weight. This level of consumption pairs well with the Asian elephant consumption reports that indicate rates of 1.5–2% of body mass per day (using the highest possible needs to take into account non-optimal land and conservation status)^45^.

We use the North Siberian estimated body mass for the woolly mammoth, which results in a daily requirement of 78–104 kg of forage day^−1^ or 28–38 tonnes year^−1^. This is consistent with previous estimates that woolly mammoths consumed roughly 118–140 kg/day of wet biomass to reach daily nutritional requirements^60^.

### Study area

We focused on ecoregions of Northern Alaska due to its relatively well-studied physiographic qualities. The area consists primarily of the permafrost zones north of the Arctic Circle including the Beaufort Coastal Plain, Brooks Foothills, Northern Brooks Range, and Southern Brooks Range. Some extensions in coverage reach into Kotzebue Sound Lowlands, Kobuk Ridges and Valleys, Davidson Mountains, Yukon Old Crow Basin, and North Ogilvie Mountains. This area benefits from extensive geological and ecological mapping conducted by Jorgenson et al.^107^ (Supplementary Fig. 2).

Permafrost is relatively continuous throughout the region at depths from 200 to 600 m while the active layer depth can vary significantly based on local conditions (which ranges from 0 to 30 m; see Supplementary Table 2)^108,109^. The ecoregions exhibit a broadly latitudinal banding pattern that consists of Arctic coastal plains and uplands in the north, transitional foothills and mountain ranges in the middle, onto boreal ecosystems toward the southern section (Supplementary Fig. 2). In parallel, vegetation assemblages range from lowland tundra dominated by sedges and small shrubs in the northern section, shrub tundra in the foothills section, and onto boreal forests south of the tree line^110^.

### Regional biomass carbon density estimates

Since the relationship between satellite-derived spectra and biomass is not straightforward at fine resolution^111^, we chose to use a recent meta-analysis-guided global biomass carbon density dataset, leveraging ORNL DAAC’s Global Aboveground and Belowground Biomass Carbon Density Maps for the Year 2010 to generate biomass carbon density estimates at 300 m resolution in Northern Alaska and the Seward Peninsula^112^.

Aboveground living biomass carbon density includes carbon stored in living plant tissues located above the earth’s surface (stems, bark, branches, twigs) but does not include leaf litter or coarse woody debris that was once attached to living plants but have since been deposited and are no longer living.

### Downgrading steps to account for preferred forage, digestibility rates, and forage utilization rate

To evaluate ecosystem support for a potential woolly mammoth population in Northern Alaska, we sought to estimate digestible forage in the area.

We downgrade estimates of aboveground biomass with preferred forage types, digestibility rates, and forage/utilization rates to form a lower, conservative estimate for aboveground digestible biomass (AgDB) for a woolly mammoth.

We chose to base biomass estimates on the Global Aboveground Biomass Carbon Density Maps for the year 2010 due to its potential global applicability, its reliance on an updated Arctic-specific model for North Alaska that accounts for shrub biomass^17^, usefulness for multiple vegetation types, and reported lack of systemic bias^112,113^.

#### Preferred forage types

We derived the lower bound of biomass estimates from the cumulative standard errors reported in the global dataset (based on the 95% prediction interval and, since the dataset represented carbon-based, not total, biomass, converting it back to total biomass by multiplying it by a factor of 2.03).

Using 2020 plant functional top cover estimates from NASA’s ABoVE: Modeled Top Cover by Plant Functional Type over Alaska and Yukon, 1985–2020 dataset, we downgraded the biomass measures to reflect the potentially preferred diet of woolly mammoths by only including the biomass per km^2^ of (1) all forbs, (2) all graminoids, and (3) all deciduous shrubs, prior to the downgrading steps^114^.

While lichen, moss, evergreen shrubs and trees could provide potential forage, we omitted these functional types to maintain a conservative approach and focus on likely preferred forage.

All raster datasets were aggregated to 300 m spatial resolution.

Modelled values for percent cover (%) for each plant functional type were used as a downgrading factor in lieu of a lower bound because the latter (estimated based on the RMSE) resulted in values outside the range of possibility (0–100%).

#### Digestibility rates

Each plant functional type was further downgraded to the digestible percentage based on reported digestibility factors of herbivores spanning digestion types, including caribou (ruminants), muskoxen (ruminants), and horses (monogastric non-ruminants like the woolly mammoth) in Arctic biomes.

Studies on arctic herbivore digestibility report factors of approximately ~ 0.8 for forbs, ~ 0.5 for graminoids (grasses, sedges, and rushes), and ~ 0.6 for deciduous shrubs^94,96,115,116^. Shrubs were downgraded by a further 50% to adjust for likely proboscidean dietary preferences and to avoid biasing the overall availability toward the more abundant shrub biomass (approximately 50% of plant biomass in the area).

#### Forage/utilization rates

Grazing intensity estimates for megafaunal grazers reportedly range from 7 to 87% (elk, bison)^117–119^, as detailed in^120^. Among cattle and bison, conservative grazing intensity estimates use 25% utilization on grass and 12.5% utilization on wet meadows^121^.

Keeping with conservative estimates, we use the lowest reported forage rate of ~ 10% applied to the new total aboveground biomass of the three preferred plant functional types to adjust for sustainable consumption, ensure low competition with the existing guild of herbivores, and account for unknowns in forage palatability.

Extant wild herbivore guilds have been shown to consume a median of 11% of net primary productivity (NPP)^52^. Further, the study estimates that the consumption would rise to 21% if the full complement of Late Pleistocene terrestrial mammals were present, indicating the scalar effects of extinct megaherbivores to the defaunated zones. This is relatively aligned with our estimated forage rate.

### Regional downgraded estimates based on the relationship between total aboveground biomass and net primary productivity

Since woolly mammoths will only eat that which was grown the same year, we further downgraded the aboveground biomass estimates to take into account the forage which only grew within the last year (primarily summer) for consumption. We chose not to use a global NPP database due to the errors inherent to transforming raw satellite data to heterogeneous/seasonal landscape metrics^122–125^. We instead opted to use an additional downgrading factor to convert total biomass to annually generated biomass estimates. To this end we leveraged a recent study referencing a set of representative field sites commonly used for arctic calibration along a North–South transect. The data are derived from a modeling framework that uses field-based parameters to predict various vegetation properties in the changing Arctic climate. Averaging the estimates from the third table in Euskirchen et al. (tussock and wet sedge representing graminoids; heath and other shrubs representing shrubs, forbs assumed to be similar), working under the assumption that all values are aboveground estimates, C-based biomass and NPP are found to be related by a ratio of 0.4^126^. We thus derived a crude, Arctic-specific conversion factor of 0.4 between carbon-based biomass (g C m^−2^) to NPP (g C m^−2^y^−2^). Since carbon-based and total biomass are linearly related, this conversion factor of 0.4 was therefore applied to the relationship as well between mean digestible biomass (Mg km^−2^) and mean digestible annually generated biomass (Mg km^−2^); see Supplementary Table 2 for details.

### Sensitivity analysis

Since our model inherently relies on numerous assumptions that cannot be further refined with available data, we performed a sensitivity analysis assessing the variability in woolly mammoth (WM) densities calculated using (1) mean, lower and upper confidence limits (LCL/UCL) of the base aboveground biomass data from Spawn et al. (2020), and (2) lower/higher forage rates (as detailed in Supplementary Table 3).

### Supplementary Information

Supplementary Information.

## Data Availability

Raw data used in this manuscript is available at -https://doi.org/10.3334/ORNLDAAC/1763 (ORNL DAAC’s Global Aboveground and Belowground Biomass Carbon Density Maps for the Year 2010 at 300 m resolution in Northern Alaska and the Seward Peninsula), -https://doi.org/10.3334/ORNLDAAC/2032 (ORNL DAAC’s ABoVE: Modeled Top Cover by Plant Functional Type over Alaska and Yukon, 1985–2020), and-https://doi.org/10.3334/ORNLDAAC/1359 (Land Cover and Ecosystem Map Collection for Northern Alaska). In addition, raster data representing the downgraded data using digestibility factors and top cover percentage is available at https://github.com/clssl/Aboveground-Biomass, where the “r_gith_agdb2” file is an Esri Grid dataset at 300 m resolution for aboveground digestible biomass in Mg km^−2^ used for estimates. All operations were conducted in ArcGIS 10.3.1 using Map Algebra/Raster Calculator on base data and tabulated using Zonal Statistics.
