# Supplementary material for: Assessing contemporary Arctic habitat availability for a woolly mammoth proxy
Source: Sci Rep. 2024 Apr 29;14:9804. doi: 10.1038/s41598-024-60442-7 (PMC11058768; doi:10.1038/s41598-024-60442-7)
Supplement: Supplementary file 1 — Supplementary Information. [file 41598_2024_60442_MOESM1_ESM.docx]

**SUPPLEMENTARY RESULTS**

**Supplementary Table 1**. Preferred forage types of woolly mammoths (*M. primigenius*) from select independent studies/lines of evidence. Data span all *M. primigenius* specimens irrespective of latitude/habitat.

| SOURCE OF EVIDENCE: analysis method \| PREFERRED FORAGE TYPE | **Forbs** | **Graminoids** | **Deciduous shrubs** | **Trees** | **Mosses** | **Lichen** | **Green algae** |
| --- | --- | --- | --- | --- | --- | --- | --- |
| GUT CONTENT: gas chromatography/mass spectrometry, thermally assisted hydrolysis and methylation, and DNA sequencing |  | -grasses  -sedges ^1^ | -shrubs ^1^  -dwarf willow (*Salix*) twigs ^1^ | -alder (*Almus*) twigs  -birch (*Betula*) twigs  -larch (*Larix*) twigs  -spruce (*Picea*) twigs ^1^ | -mosses ^1^ |  |  |
| GUT CONTENT: visual inspection |  |  |  |  | -mosses ^2^ |  |  |
| FAT CONTENT: fatty acid profiling |  |  |  |  | -mosses ^3^ | -lichens characteristic of the Siberian tundra (*Cladina arbuscula, Cladina stellaris, Leptogium saturninum*) ^3^ |  |
| GUT CONTENT: palynological analyses |  | -grasses (19.4% of gut content) ^4^ | -shrubs, undershrubs, & trees (3.6% of gut contents) ^4^ | -shrubs, undershrubs, & trees (3.6% of gut contents) ^4^ | -mosses ^4^ |  |  |
| GUT CONTENT: meta-proteomic analyses by shotgun mass spectrometry | -small flowering plants (*Arabidopsis thaliana*) ^5^  -small flowering plants (*Oxytropis sordida* of the *Pailionoideae* family) ^5^ |  | -large shrubs/small trees (*Prunus padus*) ^5^ | -large shrubs/small trees (*Prunus padus*) ^5^ | - mosses (*Huperzia* genus) ^5^ |  | -freshwater green algae from drinking (*Tetradesmus obliqus, Chaetosphaeridium globosum*) ^5^ |
| GUT AND COPROLITE CONTENT/SAMPLES: DNA metabarcoding | -forbs, dominantly ^6^ |  | -trees and shrubs (trace amounts) ^6^ | -trees and shrubs (trace amounts) ^6^ |  |  |  |
| GUT CONTENT: DNA metabarcoding, palynological, and macrofossil analyses | -forbs (*Anemone patens, Myosotis alpestris, Chamaenerion angustifolium*) ^7^ | -graminoids, dominantly (*Puccinellia* species, *Deschampsia cespitosa, Alopecurus magelanicus*, *Carex* species, *Eriophorum* species) ^7^ | -shrubs (*Salix*) (small amounts) ^7^ | -deciduous trees (small amounts) ^7^ | -mosses (*Polytrichastrum alpinum*) ^7^ |  |  |
| TOOTH ENAMEL: isotopic analyses |  | -C4 grasses, dominantly ^8^  -C3 grasses ^9^ | -C3 shrubs and trees if needed ^8^  -C3 shrubs and trees ^9^ | -C3 shrubs and trees if needed ^8^  -C3 shrubs and trees ^9^ |  |  |  |


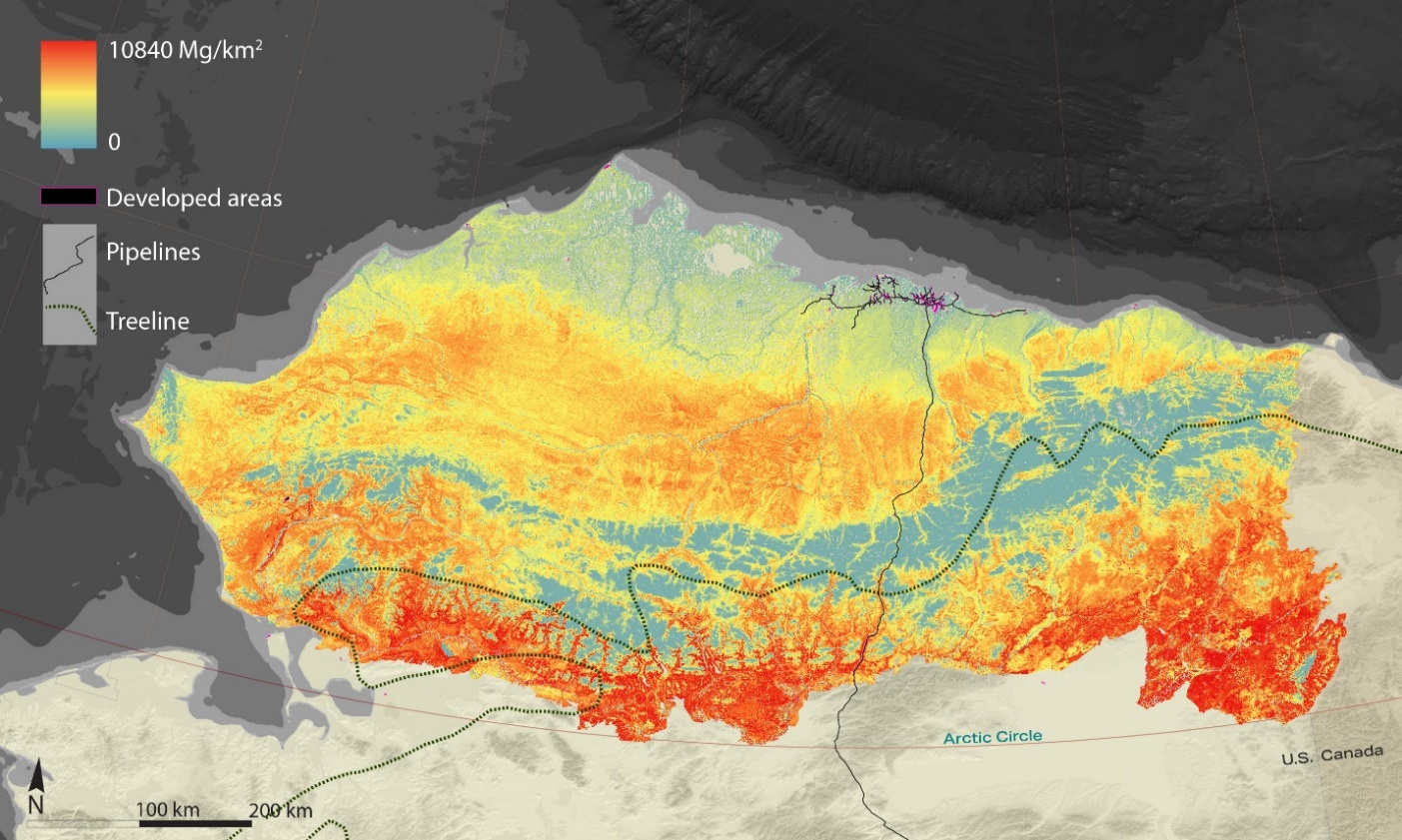


**Supplementary Figure 1**. Total aboveground dry vegetation biomass in the North Slope of Alaska. Map created in Esri ArcGIS 10.3.1 (<https://support.esri.com/en-us/products/arcmap>) and text/legends in Adobe Illustrator 28.3 (<https://www.adobe.com/products/illustrator.html)>.


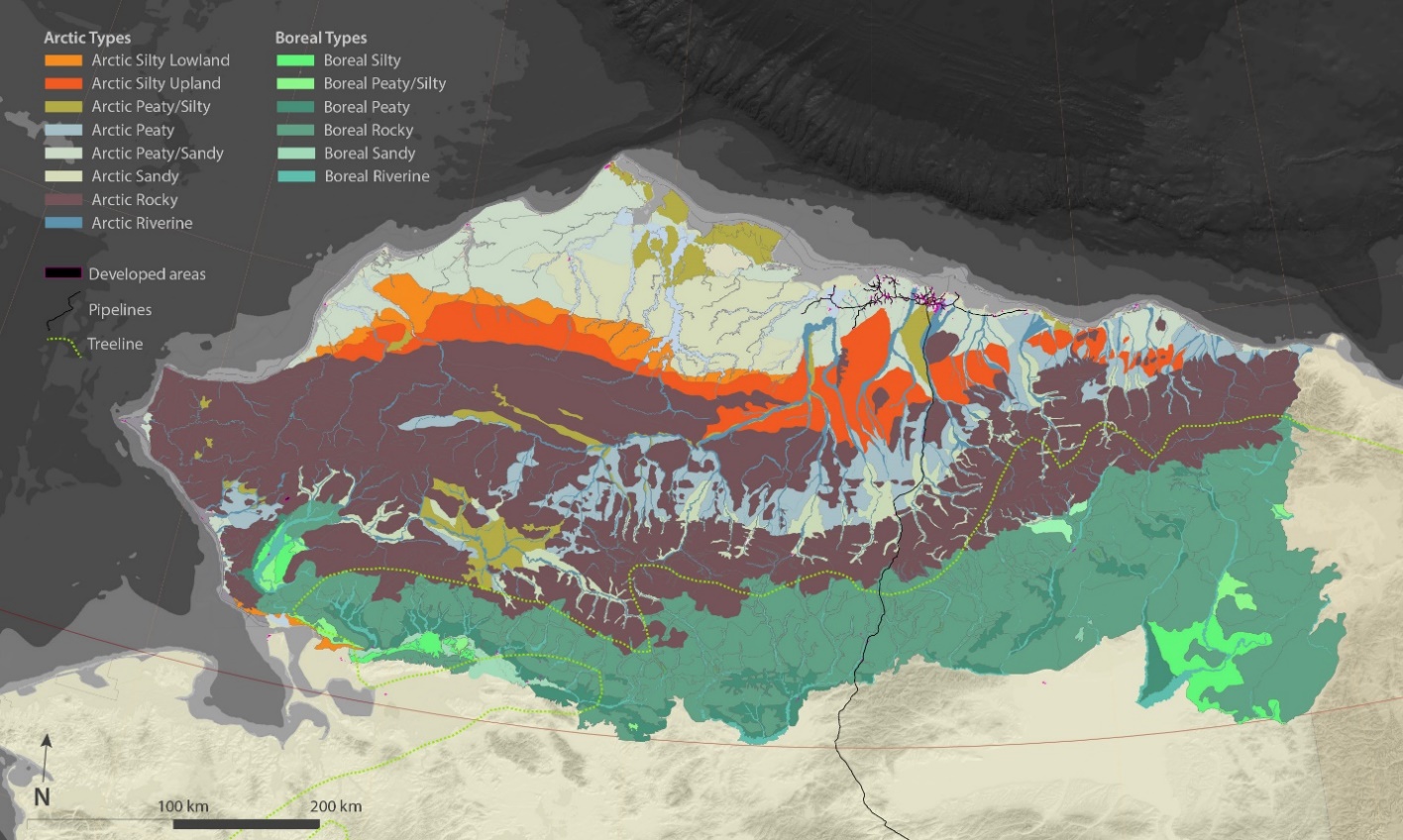


**Supplementary Figure 2**. Distribution of Arctic and Boreal ecological landscapes in the North Slope of Alaska as defined by Jorgenson et al ^10^. Map created in Esri ArcGIS 10.3.1 (<https://support.esri.com/en-us/products/arcmap>) and text/legends in Adobe Illustrator 28.3 (<https://www.adobe.com/products/illustrator.html)>.

**Supplementary Table 2**. Variables relating the ecological landscapes in the North Slope of Alaska as defined by Jorgenson ^10^ to woolly mammoth carrying capacity estimates. The Massive Ice (%) represents the percentage by volume of ice (mostly in the form of ice wedges), based on ground ice associated with generalized geology, in each of the ecological landscape type ^10^. The Maximum Potential Thaw Settlement, based on ground ice associated with generalized geology, represents the maximum increase in the depth of seasonally thawed soil (the active layer) ^10^. The magnitude of thaw settlement is related to the nature and abundance of ground ice as well as the severity of a natural or anthropogenic disturbance. Massive Ice (%) and Maximum Potential Thaw Settlements together thus reflect the vulnerability of ice-rich permafrost to thawing. The MAAT (°C) represents the mean air average temperature. The Mean Digestible Biomass Density (Mg km^-2^) represents the total density of the downgraded biomass of the three selected preferred plant functional types (forbs, graminoids, deciduous shrubs). The Mean Digestible Annually Generated Biomass (Mg km^-2^) represents the estimated annually generated downgraded biomass of the three selected preferred plant functional types (forbs, graminoids, deciduous shrubs), related to the Mean Digestible Biomass Density by an estimated factor of 0.4 (see main text’s Methods for details). The Area (km^-2^) estimates represent the total area of each ecological landscape type ^10^. The High Estimated Woolly Mammoth Density (woolly mammoth km^-2^) represents the higher bound density based on a lower estimated woolly mammoth body mass (of 3.9 tonnes), while the Low Estimated Woolly Mammoth Density (woolly mammoth km^-2^) represents the lower bound density based on a higher estimated woolly mammoth body mass (of 5.2 tonnes).

| **Ecological Landscape Type** | **Massive Ice (%)** | **Maximum Potential Thaw Settlement (meters)** | **MAAT (°C)** | **Mean Digestible Biomass Density (Mg km^-2^)** | **Annually Generated Mean Digestible Biomass Density**  **(Mg km^-2^)** | **Area (km^2^)** | **High Estimated Woolly Mammoth Density (woolly mammoth km^-2^) (for 3.9 tonnes/ woolly mammoth)** | **Low Estimated Woolly Mammoth Density (woolly mammoth km^-2^) (for 5.2 tonnes/ woolly mammoth)** |
| --- | --- | --- | --- | --- | --- | --- | --- | --- |
| Boreal Sandy Riverine | <5% | <0.5 | -5 | 274 | 109,6 | 934 | 0,38 | 0,29 |
| Boreal Rocky Upland | 5-10% | 0-10 | -7 | 202 | 80,8 | 29888 | 0,28 | 0,21 |
| Boreal Silty Lowland | 30-70% | 5-30 | -6 | 193 | 77,2 | 5797 | 0,27 | 0,20 |
| Boreal Peaty Glaciated Upland | 10-80% | 5-30 | -6 | 177 | 70,8 | 10810 | 0,25 | 0,19 |
| Boreal Rocky Glaciated Upland | 10-80% | 5-30 | -8 | 163 | 65,2 | 11886 | 0,23 | 0,17 |
| Boreal Rocky Circum. Alpine | <5% | <0.5 | -8 | 163 | 65,2 | 1147 | 0,23 | 0,17 |
| Boreal Gravelly Riverine | <5% | <0.5 | -7 | 153 | 61,2 | 5831 | 0,21 | 0,16 |
| Boreal Rocky Ultramafic Alpine | <5% | <0.5 | -6 | 146 | 58,4 | 84 | 0,20 | 0,15 |
| Boreal Rocky Acidic Alpine | <5% | 0.5-2 | -9 | 138 | 55,2 | 33531 | 0,19 | 0,15 |
| Boreal Peaty Silty Lowland | 5-30% | 2-5 | -6 | 137 | 54,8 | 1860 | 0,19 | 0,14 |
| Arctic Rocky Upland | 5-10% | 0.5-2 | -10 | 131 | 52,4 | 50335 | 0,18 | 0,14 |
| Boreal Peaty Gravelly Lowland | 5-10% | 0.5-2 | -7 | 130 | 52 | 1190 | 0,18 | 0,14 |
| Arctic Peaty Glaciated Upland | 10-80% | 5-30 | -10 | 117 | 46,8 | 11708 | 0,16 | 0,12 |
| Arctic Peaty Gravelly Lowland | 5-30% | 0.5-5 | -11 | 108 | 43,2 | 8943 | 0,15 | 0,11 |
| Arctic Silty Upland | 30-70% | 0.5-30 | -12 | 106 | 42,4 | 21446 | 0,15 | 0,11 |
| Boreal Rocky Alkaline Alpine | <5% | <0.5 | -9 | 103 | 41,2 | 15436 | 0,14 | 0,11 |
| Arctic Rocky Glaciated Upland | 10-80% | 5-30 | -10 | 96,5 | 38,6 | 13405 | 0,13 | 0,10 |
| Arctic Silty Lowland | 30-70% | 5-30 | -11 | 88,8 | 35,52 | 7083 | 0,12 | 0,09 |
| Arctic Rocky Acidic-Alk. Alpine | <5% | 0.5-2 | -10 | 75,8 | 30,32 | 3879 | 0,11 | 0,08 |
| Boreal Sandy Upland | <5% | <0.5 | -5 | 68,3 | 27,32 | 713 | 0,10 | 0,07 |
| Arctic Peaty Silty Lowland | 10-80% | 2-30 | -11 | 67,4 | 26,96 | 10898 | 0,09 | 0,07 |
| Arctic Gravelly Riverine | <5% | <0.5 | -10 | 61,5 | 24,6 | 9210 | 0,09 | 0,06 |
| Arctic Rocky Acidic Alpine | <5% | 0.5-2 | -10 | 57,5 | 23 | 56713 | 0,08 | 0,06 |
| Boreal Rocky Acidic-Alk. Alpine | <5% | 0.5-2 | -10 | 55,5 | 22,2 | 596 | 0,08 | 0,06 |
| Arctic Peaty Sandy Lowland | 10-30% | 0.5-5 | -11 | 51,8 | 20,72 | 27216 | 0,07 | 0,05 |
| Arctic Sandy Lowland | 5-10% | <0.5 | -12 | 37,5 | 15 | 13956 | 0,05 | 0,04 |
| Arctic Sandy Riverine | <5% | <0.5 | -12 | 35,6 | 14,24 | 3693 | 0,05 | 0,04 |
| Arctic Peaty Silty Riverine | 10-30% | 2-5 | -12 | 32,8 | 13,12 | 1166 | 0,05 | 0,03 |
| Arctic Rocky Circum. Alpine | <5% | <0.5 | -11 | 30,6 | 12,24 | 827 | 0,04 | 0,03 |
| Arctic Gravelly Coast | <5% | <0.5 | -7 | 29,5 | 11,8 | 637 | 0,04 | 0,03 |
| Arctic Rocky Ultramafic Alpine | <5% | <0.5 | -9 | 22,1 | 8,84 | 1304 | 0,03 | 0,02 |
| Arctic Rocky Alkaline Alpine | <5% | <0.5 | -10 | 16,8 | 6,72 | 20716 | 0,02 | 0,02 |
| Arctic Sandy Coast | <5% | <0.5 | -12 | 13,4 | 5,36 | 974 | 0,02 | 0,01 |
| Arctic Coastal Water | 0 | <0.5 | -11 | 1,93 | 0,772 | 7202 | 0,00 | 0,00 |
| Arctic Freshwater | 0 | 0 | -12 | 0,53 | 0,212 | 891 | 0,00 | 0,00 |
| Boreal Freshwater | 0 | 0 | -8 | 0,45 | 0,18 | 99 | 0,00 | 0,00 |
| Arctic Marine Water | 0 | 0 | -11 | 0 | 0 | 21069 | 0,00 | 0,00 |

**Supplementary Table 3.** Sensitivity analysis assessing the variability in woolly mammoth densities calculated using (1) mean, lower and upper confidence limits (labeled LCL and UCL, respectively) of the base aboveground biomass data from Spawn et al. ^11^, and (2) low, medium, and high forage rates. The lower bound for each estimate is 0 due to the fact that various Arctic landscape units (e.g. Arctic Sandy Coast) have minimal biomass and could not support any woolly mammoths. The entire dataset indicates a mean tendency for 0-0.65 woolly mammoth km^-2^.

| **Base Aboveground Digestible**  **Biomass Test Level** | **Highest Estimated Woolly Mammoth Density (woolly mammoth km^-2^) (for a 3.9 tonne woolly mammoth)** | | |
| --- | --- | --- | --- |
|  | **Low Forage Rate (5%)** | **Med. Forage Rate (10%)** | **High Forage Rate (20%)** |
| **LCL** | 0.19 | 0.38 | 0.77 |
| **Mean** | 0.32 | 0.65 | 1.3 |
| **UCL** | 0.67 | 1.3 | 2.7 |

**References**

1. van Geel, B. *et al.* The ecological implications of a Yakutian mammoth’s last meal. *Quat. Res.* (2008). doi:10.1016/j.yqres.2008.02.004

2. Fisher, D. C. *et al.* Anatomy, death, and preservation of a woolly mammoth (Mammuthus primigenius) calf, Yamal Peninsula, northwest Siberia. *Quat. Int.* (2012). doi:10.1016/j.quaint.2011.05.040

3. Guil-Guerrero, J. L. *et al.* The fat from frozen mammals reveals sources of essential fatty acids suitable for palaeolithic and neolithic humans. *PLoS One* (2014). doi:10.1371/journal.pone.0084480

4. Ukraintseva, V. V. Vegetation of warm late pleistocene intervals and the extinction of some large herbivorous mammals. *Polar Geogr. Geol.* (1981). doi:10.1080/10889378109388689

5. Cucina, A. *et al.* Meta-proteomic analysis of the Shandrin mammoth by EVA technology and high-resolution mass spectrometry: what is its gut microbiota telling us? *Amino Acids* (2021). doi:10.1007/s00726-021-03061-0

6. Willerslev, E. *et al.* Fifty thousand years of Arctic vegetation and megafaunal diet. *Nature* (2014). doi:10.1038/nature12921

7. Polling, M. *et al.* Multiproxy analysis of permafrost preserved faeces provides an unprecedented insight into the diets and habitats of extinct and extant megafauna. *Quat. Sci. Rev.* (2021). doi:10.1016/j.quascirev.2021.107084

8. Metcalfe, J. Z., Longstaffe, F. J., Ballenger, J. A. M. & Vance Haynes, C. Isotopic paleoecology of Clovis mammoths from Arizona. *Proc. Natl. Acad. Sci. U. S. A.* (2011). doi:10.1073/pnas.1113881108

9. Widga, C. *et al.* Life histories and niche dynamics in late Quaternary proboscideans from midwestern North America. *Quat. Res. (United States)* (2021). doi:10.1017/qua.2020.85

10. Jorgenson, M. T. *et al.* Permafrost database development, characterization, and mapping for northern Alaska. *U.S. Fish Wildl. Serv.* (2014).

11. Spawn, S. A. & Gibbs, H. K. Global Aboveground and Belowground Biomass Carbon Density Maps for the Year 2010. *Ornl Daac* (2020).
